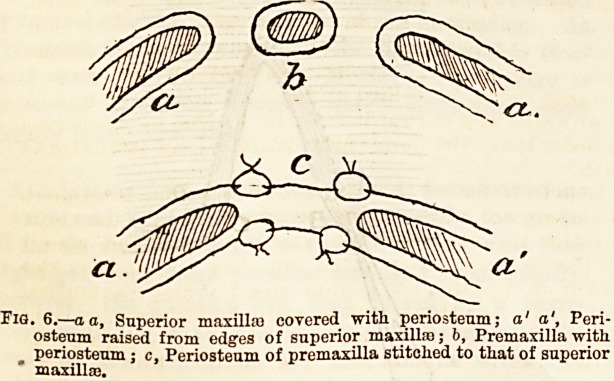# The Treatment of Hare Lip

**Published:** 1893-11-04

**Authors:** Alex. Miles


### The Hospital Clinic.

[*The Editor will be glad to receive offers of co-operation and contributions from members of the profession. All letters should be addressed to* The Editor, The Lodge, Porchester Square, London, W.]

### ROYAL INFIRMARY, EDINBURGH.

### The Treatment of Hare-Lip.

The congenital deformity known as hare-lip consists in a cleft, more or less complete, in the substance of the upper lip. Inasmuch as this fissure is never placed mesially, the comparison of the condition with that existing in the hare is not strictly accurate. At an early stage in development the central part of the upper lip is represented by a flap of tissue attached to the nose and frontal region—the fronto-nasal plate—and the lateral portions are derived from the coverings of the upper jaw, which fuse with the central portion at a later date. Sometimes this union fails to take place on one side only, sometimes on both.

The condition is not uncommon, and is one which offers considerable hope of benefit from operation, provided the sufferer is in moderately good health when brought under the surgeon's notice, and that all the necessary precautions are observed. The repair of hare-lip, in itself a comparatively simple matter, is one of those operations the success of which depends entirely on careful attention to the minutest details.

Fig. 1.

A considerable number of the children presented to the surgeon for this condition are weakly, ill-nourished creatures, suffering from diarrhœa, sickness, &c., but the greater number are in perfect health. Those of the latter class should never be denied the advantages of operation. Apart from merely æsthetic considerations, the repair of the cleft in the lip improves the child's power of sucking, and so favours its nutrition, and in addition prevents any faulty articulation when it begins to speak.

Authorities differ as to the age at which the operation should be performed. Mr. Joseph Bell, who has had great experience, and as great success in the operation, considers that in strong children it may best be undertaken between the second month and the beginning of the first dentition—about the seventh month; while in weakly subjects the fifth month is early enough to begin treatment. Should the operation have been delayed till after dentition has commenced it is advisable to do nothing till it is over.

As the operation, in most cases, is undertaken with the main object of removing an unsightly deformity, every care must be taken to do this completely; and to avoid everything which, by leading to non-union of the edges, will leave the patient worse than he was found. The main risks of failure lie in the following directions, and are more or less preventable.

(1) Breaking down of the wound, which may be due to various causes, such as the child forcibly disturbing the dressing with its hands; carelessness and clumsiness on the part of the nurse feeding the child; crying or coughing; and last of all sepsis preventing primary union. The surgeon should avoid the last by arresting all bleeding before bringing the rawed edges together, by careful suturing and by the use of an anti-

Fig. 2.—*a*, Cleft with line of curved incisions; *b*, After insertion of stitches; *c*, Ultimate result, slight papilla.

septic wash for the mouth and lips, which is conveniently applied on a camel's-hair brush. The prevention of the other causes is chiefly in the hands of the nurse, who should, if possible, be known to the child.

(2) A symmetrical union. As the two sides of the cleft are often unequal in length it requires some care to bring the edges together so as to correct this. By passing the lowest stitch through the longer flap obliquely from below upwards, and then entering the shorter flap at a slightly higher level, the two may be brought into apposition (Fig. 1).

(3) Sometimes the stitches or pins by which the edges of the wound are kept in contact, are kept in too long, and marks are left. By removing the deep, external stitches at the end of forty-eight hours or earlier this will be obviated.

(4) Another source of partial failure is the leaving of a small notch at the free margin of the lip. To avoid

Fig. 3.

this various devices have been had recourse to. One of the simplest is that of the late Professor Spence, who pared the edges of the cleft by two curved incisions (Fig. 2) with their concavities towards each other, so that when brought together there was a redundency of tissue, which formed a small papilla after contraction had taken place during healing.

Professor Chiene has for some years been employing another method of dealing with hare-lip. Figs. 3, 4, and 5, copied from his paper in the Edinburgh Hospitals Reports, 1893, illustrate the principle. Instead

Fig. 4.

of paring the edges of the cleft, he splits it along the junction of skin and mucous membrane, and then unites skin to skin, and mucous membrane to mucous membrane. By stopping short of the free edge of the lip he prevents the leaving of a notch. He describes the steps in the operation as follows: "It is best to lay hold of the two lower corners of the cleft with catch-forceps, then putting the edges of the cleft on the stretch with a narrow knife, transfix the edge of the cleft at A (Fig. 3) and cut towards the edge of the cleft to B. Do the same on the other side, D to C, then split the cleft from A to E, and from D to E, along the line of junction of skin with mucous membrane.

Fig. 5.

You have then two flaps of mucous membrane which are to be brought into apposition. At first I used to unite these edges with catgut, but recently I found that the edges of mucous membrane could be brought into accurate contact with horsehair sutures if these are passed from the skin surface down to the edge of mucous membrane, picking up the opposite edge, and brought out through the skin surface on the opposite side, and the ends tied together. Three of such sutures are generally sufficient (Fig. 4). Superficial intermediate stitches, also of horsehair, unite the skin surfaces accurately. Lastly, to complete the operation, the two tags which are left at the free edge of the lip

Fig. 6.—*a a*, Superior maxillæ covered with periosteum; *a' a'*, Periosteum raised from edges of superior maxillæ; *b*, Premaxilla with periosteum; *c*, Periosteum of premaxilla stitched to that of superior maxillæ.

have to be dealt with, these are united to one another by catgut sutures, taking care to leave a projection so as to prevent any tendency to the formation of an unseemly notch" (Fig. 5).

When the deformity is bilateral the operation is more difficult, and the result less satisfactory. The principle guiding the procedure is the same, but the projecting premaxillary mass requires special attention. Mr. Chiene's practice is to use the skin over it to form a nasal septum, and by means of a transverse incision through the mucous membrane and periosteum to remove the bone, leaving the periosteum and mucous membrane, which are subsequently stitched to the superior maxilla, and reproducing bone complete the alveolar arch (Fig. 6).

In all these procedures success depends chiefly on (1) taking every precaution against sepsis, (2) arresting all bleeding before inserting stitches, and (3) freely separating the lip from the alveolar margin when there is the least difficulty in approximating edges.

Alex. Miles, M.D., F.R.C.S.Edin.

## Figures and Tables

**Fig. 1. f1:**
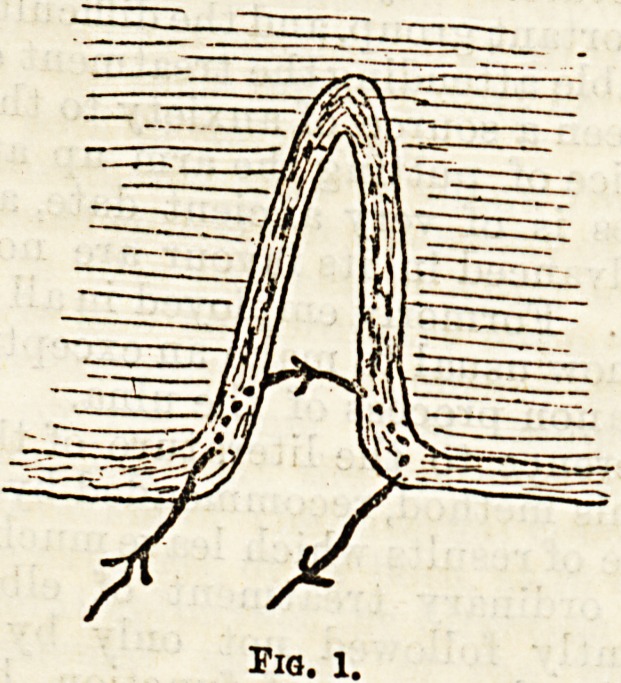


**Fig. 2. f2:**
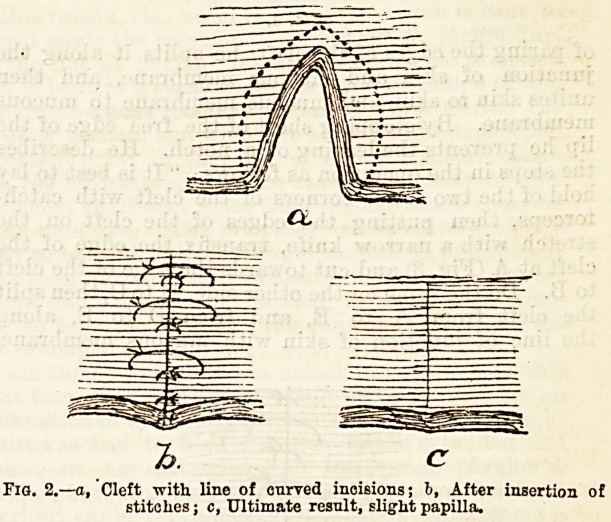


**Fig. 3. f3:**
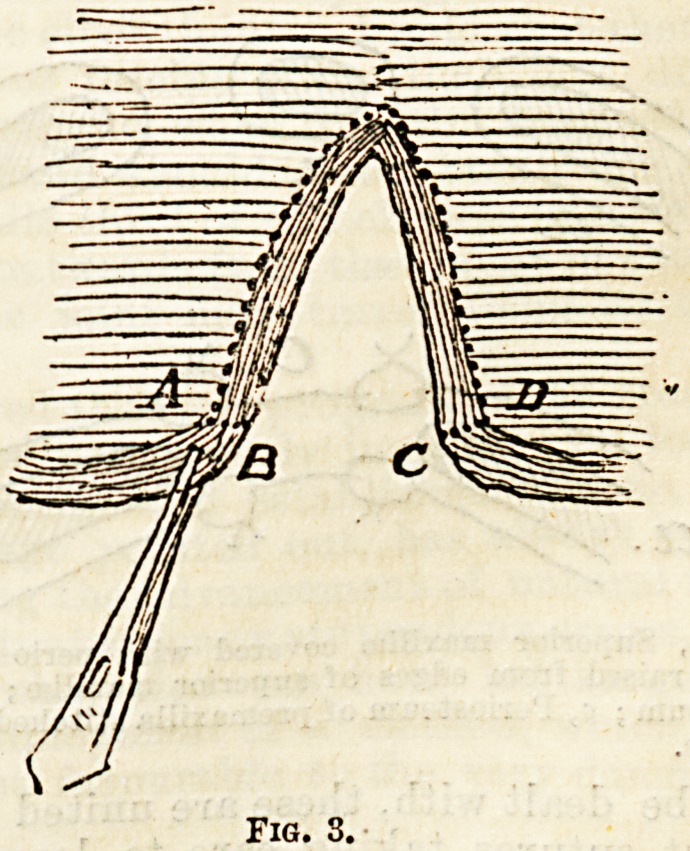


**Fig. 4. f4:**
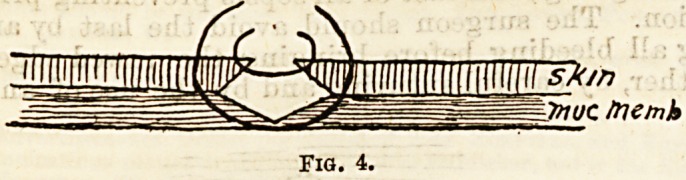


**Fig. 5. f5:**
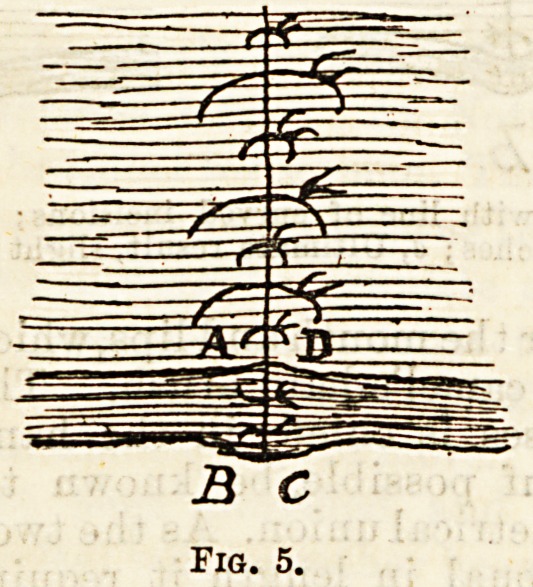


**Fig. 6. f6:**